# 48XXYY Syndrome in an Adult with Type 2 Diabetes Mellitus, Unilateral Renal Aplasia, and Pigmentary Retinitis

**DOI:** 10.1155/2010/612315

**Published:** 2010-08-17

**Authors:** Baha Zantour, Mohamed Habib Sfar, Samia Younes, Wafa Alaya, Mahdi Kamoun, Emna Mkaouar, Saida Jerbi

**Affiliations:** ^1^Department of Endocrinology and Internal Medicine, Tahar Sfar Hospital, Hiboune, Mahdia 5100, Tunisia; ^2^Department of Molecular Human Genetics, Faculty of Medicine, Avenue Majida BOULILA, Sfax 3029, Tunisia; ^3^Department of Radiology, Tahar Sfar Hospital, Hiboune, Mahdia 5100, Tunisia

## Abstract

A 45-year-old male was referred for diabetes mellitus. Clinical examination found a family history of multiple precocious deaths, strong consanguinity, personal history of seizures during childhood, small testicles, small penis, sparse body hair, long arms and legs, dysmorphic features, mental retardation, dysarthria, tremor, and mild gait ataxia. Investigations found pigmentary retinitis, metabolic syndrome, unilateral renal aplasia, and hypergonadotropic hypogonadism, and ruled out mitochondrial cytopathy and leucodystrophy. Karyotype study showed a 48XXYY chromosomal type. Renal aplasia and pigmentary retinitis have not been described in 48XXYY patients. They may be related to the chromosomal sex aneuploidy, or caused by other genetic aberrations in light of the high consanguinity rate in the patient's family.

## 1. Introduction

The 48XXYY syndrome is a rare sex chromosome aneuploidy occurring in 1/50.000 among newborns, while its frequency among institutionalized mentally retarded patients is 1/300 [1]. The first description was published by Muldal and Ockey in 1960 [2] as a variant of Klinefelter's syndrome. Ten years later, Parker et al. established this aneuploidy as a distinct clinical and genetic entity [3]. Since then, case reports of 48XXYY patients showed some physical, mental, and psychological differences from 47XXY patients, but the largest series was recently reported by Tartaglia et al. describing the characteristics of this disorder [4]. Here, we report a case of a patient with 48XXYY syndrome diagnosed at the age of 45 years in order to strengthen the particularities of this syndrome and to report features not yet described in this disease.

## 2. Observation

A 45-year-old male was referred to our endocrinology department in June 2007 for diabetes mellitus fortuitously diagnosed by preoperative exams for chronic dacryocystitis. He was followed up in gastroenterology since few months for chronic B hepatitis.

**Figure 1:**
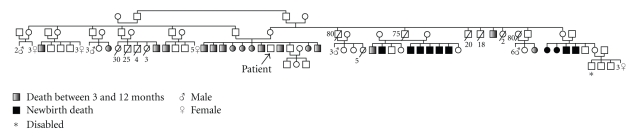
Patient's family tree. For representativeness of this large family, safe progenies are represented by female or male symbol preceded by the number of subjects. Numbers noted close to dead subjects indicate age at death.

In his family history, there was a first-degree consanguinity of patient's parents and an overall family strong consanguinity. He had a cousin with diabetes mellitus and hypertension. He was born to healthy parents. At his birth, the father, 162 cm high, was aged 40, and the mother, 148 cm high, was aged 30. So, his expected height is 161.5 cm. Our patient was the eighth of twelve children, however, he had only one brother alive, because of a family history of deaths in early stage of life among his siblings and some of his cousins. The cause of these precocious deaths was unknown, however, some of these subjects presented seizures during life (Figure 1). The only brother alive was married and had three children. Our patient was born at term of a normal pregnancy. He had a history of seizures during childhood. There was no available data about the diagnostic of these seizures, the patient received an oral drug for a little period and then his mother interrupted the treatment. He walked at 5 years and had always an awkward gait. He was able to speak only few words at 5 years. His language remained poor for several years. He had never gone to school and was unable to read and write. He was reserved, got married to his cousin two years ago, but did not have children.

**Figure 2:**
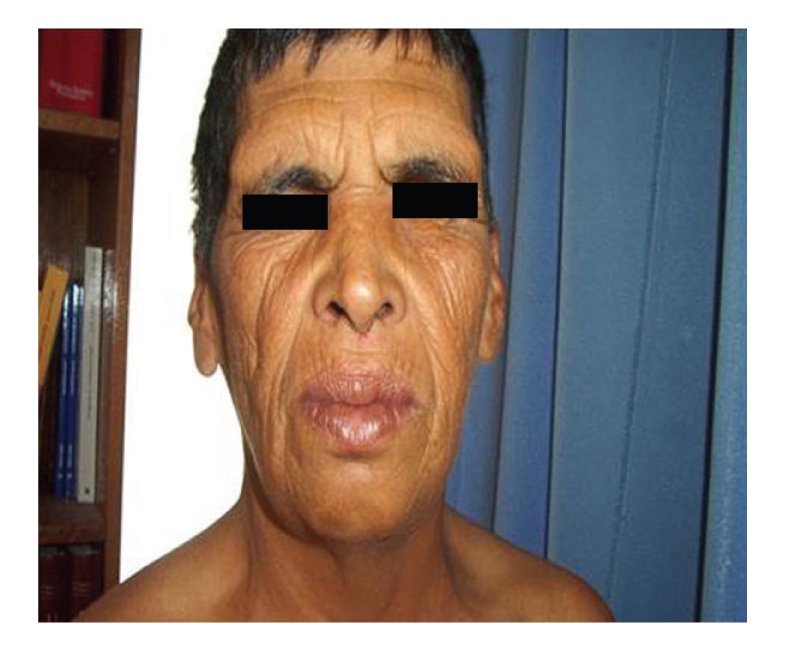
Frontal facial appearance.

**Figure 3:**
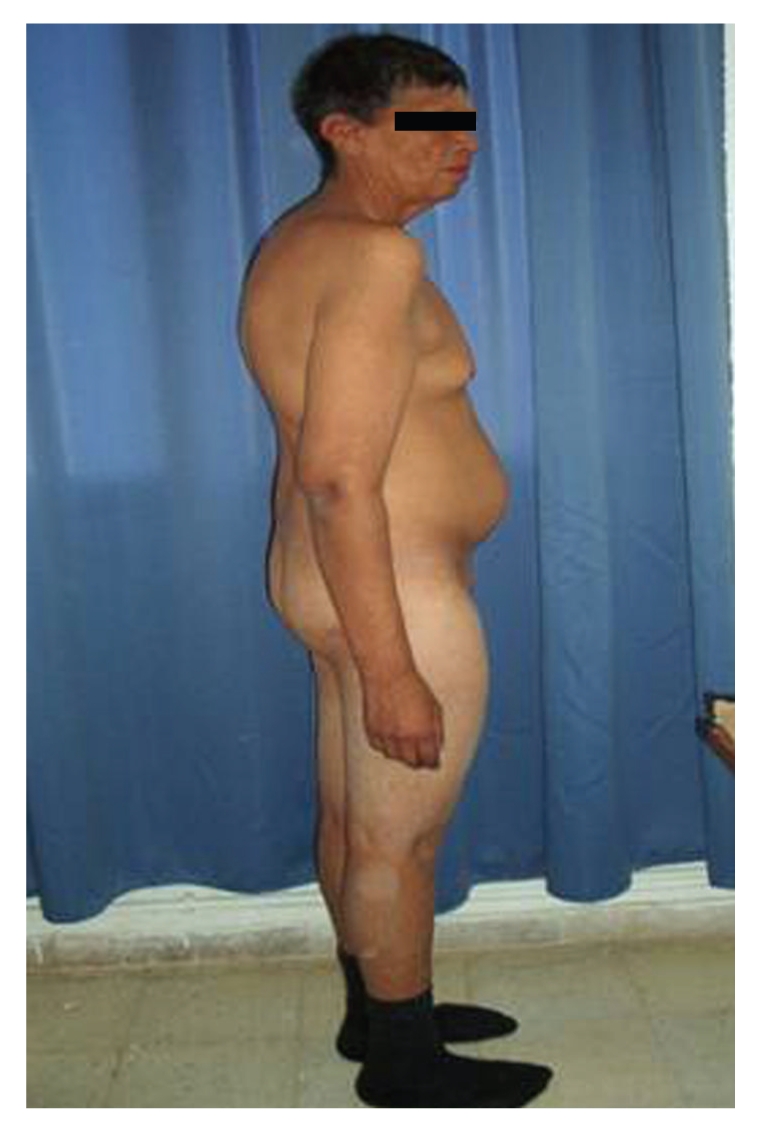
Lateral physical appearance. Note the android obesity.

**Figure 4:**
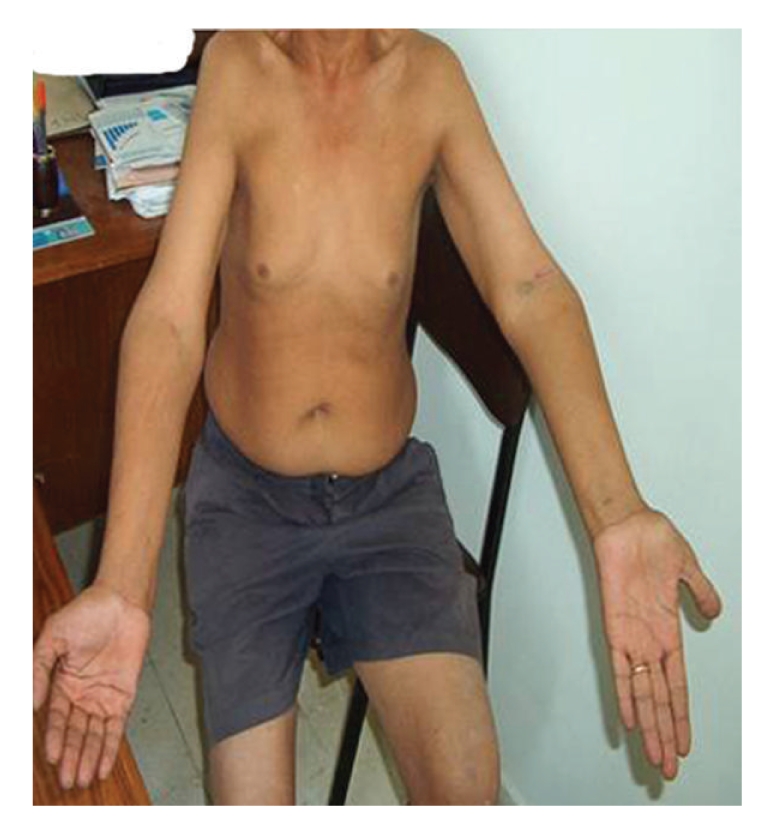
Cubitus varus of both elbows more marked in left.

Clinical examination found a 166 cm height, 75 kg weight, a body mass index 27.2 kg/m^2^, waist circumference 95 cm, hip circumference 100 cm, an increased leg and arm length (sitting height 76 cm, arm span 169 cm), and a blood pressure 120/70 cmHg. The patient had multiple dental caries and was partially edentulous. His aspect was coarse with a pugilistic facial appearance with prominent forehead and supraorbital ridges, dense scalp hair, and broad nasal root (Figures 2 and 3). He had a cubitus varus with a lack of full extension and a limitation of supination of both elbows, more marked on the left (Figure 4), small testicles measuring 1 cm in diameter, a small penis, no breast, facial nor axillary hair, with sparse pubic hair. There was no lipodystrophy, acanthosis nigricans, gynecomastia, or clinodactyly. He had postural and kinetic tremor, mild gait ataxia, dysarthria, and severe mental retardation. During his hospitalisation, we noted verbal aggressiveness when contraried.

**Figure 5:**
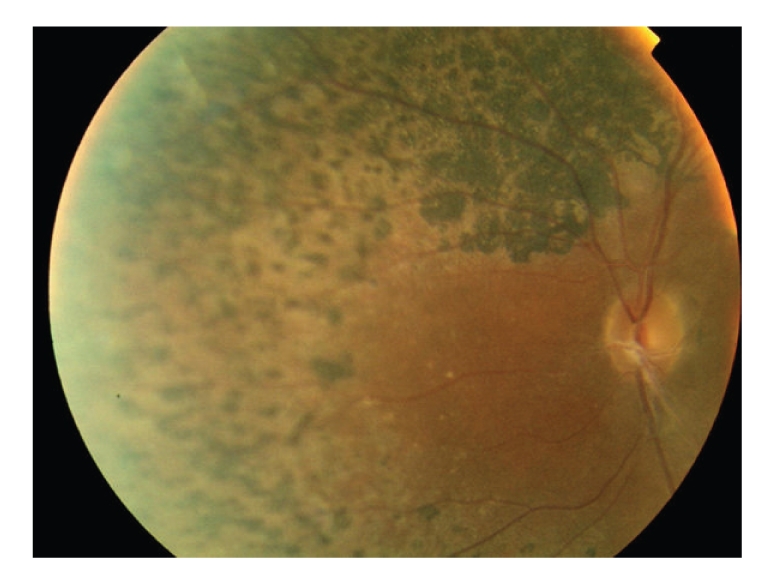
Image of the fundus showing retinitis pigmentosa: pigmentary deposits with attenuation of the retinal vessels.

**Figure 6:**
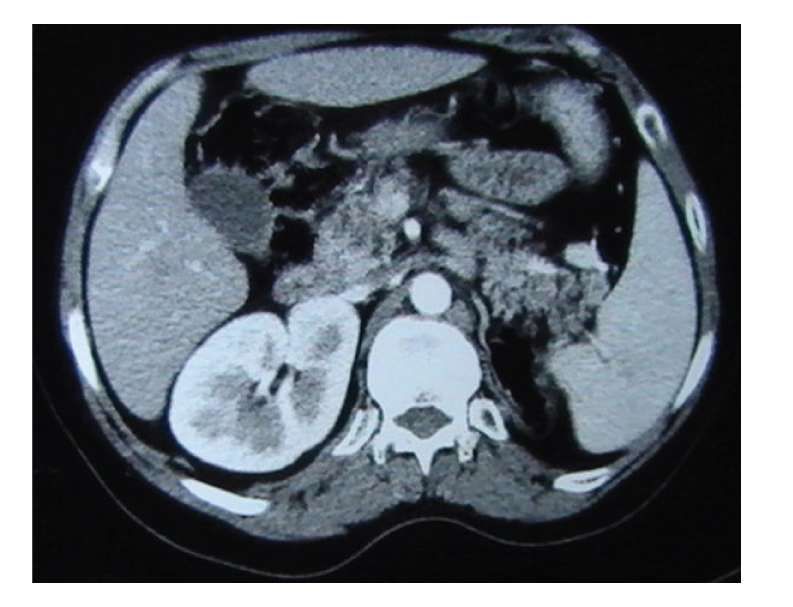
Abdominal CT: unilateral left renal aplasia.

**Figure 7:**
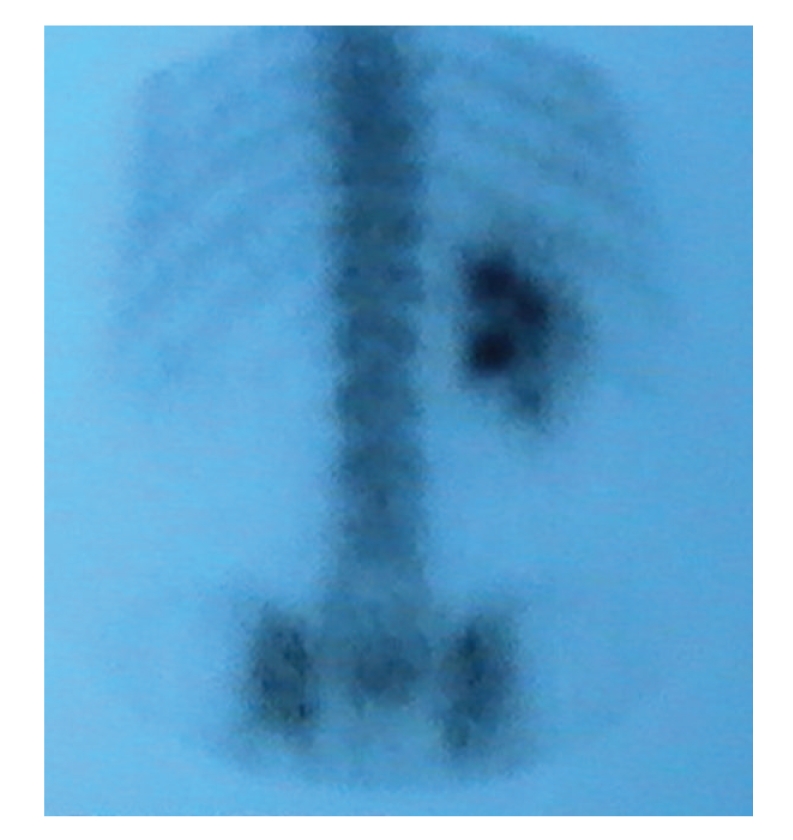
Tc-99m methylene diphosphonate scintigraphy: unilateral left renal aplasia.

 Biological exams showed hyperglycemia, increased glycated haemoglobin, decreased high density lipoproteins, cytolysis, polyclonal hypergamma-globulinemia, slightly elevated pyruvates with normal lactates (Table 1). Hormonal data showed decreased testosterone level with increased basal and stimulated gonadotrophin levels (Tables 2 and 3). Autoantibodies to glutamic acid decarboxylase, IA2, threo-peroxydase, thyroglobulin, antismooth muscle, anti-LKM1 (liver kidney microsome 1), antimitochondries and antinulear were negative. Very long chain fatty acids level was normal. Ophthalmic exam showed retinitis pigmentosa (Figure 5) and cataract. Muscular biopsy with enzymatic study, electroencephalogram, and MRI of brain were normal. Electromyogram showed signs of axonal type minor to moderate neurogenic damage of lower limbs. X-ray film of the left wrist showed radioulnar synostosis. There was a single right kidney on abdominal ultrasound, computed tomography and Tc-99m methylene diphosphonate scintigraphy (Figures 6 and 7). The liver was normal on ultrasound and computed tomography. Genetic analysis for mitochondrial DNA mutations was negative.

The karyotype, performed on lymphocytes from peripheral blood, revealed the presence of the 48XXYY aneuploidy in all the cells analyzed.

The patient was initially treated by Metformine 850 mg and Glibenclamide 5 mg twice a day, and testosterone enantate 250 mg per month. We noted an improvement of the patient's general condition, but diabetes remained uncontrolled with very high glycated haemoglobin (12% to 13%). The education for diabetes management proved to be impossible. When treated by a biphasic insulin (Mixtard30 24 IU at 8 hours, 20 IU at 20 hours) injected by a family member, we obtained a slight better control with HbA1c between 8% and 9%. Actually, the patient has a stable psychological state, he is in the phase of active HBe antigen-negative chronic B hepatitis with fluctuating cytolysis.

## 3. Discussion

The 48XXYY syndrome is now considered by most authors to be a distinct entity [4, 5]. Although its physical phenotype is similar to 47XXY, 48XXYY syndrome is associated with additional medical problems, some physical particularities and more significant neurodevelopmental and psychological features [4, 6]. Mental retardation was reported to be a constant feature in 48XXYY cases varying from nearly normal to moderate or severe. It is attributed mainly to the polysomy, and probably to a skewed X inactivation [7]. However, Tartaglia et al. [4] found only 29.1% having intellectual quotient scores within the mental retardation range. Patients with sex chromosome aneuploidies have a lag in language skills, affecting expressive language more than comprehension [8]. 48XXYY patients are more prone to have problems with aggressiveness, hyperactivity, and depression compared to males with 47XXY, resulting in daily living skills, socialization, and communication compared to other sex chromosome aneuploidies [4, 9]. Our patient had a severe mental retardation, delayed language development, dysarthria, a very limited social life, and is assisted by his mother in communicating with others. These disabilities made education for diabetes management impossible with poor control.

Hypergonadotropic hypogonadism was a constant feature in 48XXYY adult patients [4, 10, 11]. Our patient had very low testosterone levels with increased basal and stimulated levels of luteinizing hormone (LH) and follicle stimulating hormone (FSH). The ratio of LH to FSH basal levels was lower than 0.5. Stimulated levels reached levels more than 50 UL/L, suggesting dysfunction of the Leydig as well as the Sertoli cells, as described in the 48XXYY adult patients recently reported [10, 11]. However, in 47XXY adult patients, testosterone levels may be in normal range, FSH levels are usually more elevated than LH levels, indicative of a more pronounced exocrine function [12]. 

 Type 2 diabetes occurs in 18.2% of 48XXYY patients [4]. Low testosterone levels have been shown to predict type 2 diabetes and metabolic syndrome in middle-aged men [13]. There is an inverse relationship between testosterone level and obesity [14]. Hypogonadism may cause an unfavourable change in body composition, primarily through increased truncal fat and decreased muscle mass. Our patient has a type 2 diabetes with metabolic syndrome defined by an increased waist/hip circumferences more than 0.9, a decreased HDL cholesterol less than 0.4 g/L and diabetes [15]. 

Retinitis pigmentosa is a genetic disease that can be associated with mitochondrial cytopathies [16], multiple neuromuscular syndromes mainly Bardet Bledl syndrome, spinocerebellar ataxia type 7, ectodermal diseases, and some metabolic syndromes having all evocating signs, missing in our patient. Nonsyndromic retinitis pigmentosa is genetically heterogeneous. Until now, more than 10 genes were identified, and transmission can be autosomal or X-linked [17]. In our patient, we initially evocated mitochondrial cytopathy because of multiorgan anomalies associating diabetes, neuromuscular, hepatic disorders to hypogonadism, and retinitis pigmentosa, and because of family's patient history, and made necessary investigations which were negative. Personal and familial history of neurologic disorders suggested another diagnosis, the leucodystrophy, very long chain fatty acids level, and MRI of the brain were normal. Retinitis pigmentosa was not described in association with chromosome anomalies especially with sex chromosome aneuploidies.

Some physical features, such as pugilistic facial appearance, dental defects, and radioulnar synostosis were described in 48XXYY and other rare sex chromosome aneuploidies [10, 18]. Congenital heart defects are frequent in Klinefelter syndrome [19], and occur in 19.4% of 48XXYY patients [4]. Congenital renal aplasia was not described in 48XXYY patients although it was reported in Klinefelter syndrome or in other sex chromosomal polysomies [18]. The gene dose effects of noninactivated genes on the extra chromosomes may be a plausible cause of the congenital malformations and may also account for the increased risk of delayed speech, learning difficulties, and psychiatric diseases [19].

In our patient, it is possible that the retinitis pigmentosa and the renal aplasia are due to the sex chromosomal aberration. However, it cannot be excluded that the high consanguinity rate in the patient's family gave rise to homozygosity of aberrant alleles that are located on chromosomes other than the sex chromosomes.

 The parental origin of the additional sex chromosomes is not well established in 48XXYY patients. These can be paternally derived, resulting from nondisjunction at the first and second meiotic division during spermatogenesis [10, 20]. One can speculate that the additional X chromosome can be maternally derived, resulting from either meiotic nondisjunction during gametogenesis, or from mitotic nondisjunction in the developing zygote, as in 47XXY patients [21]. The patient's family history of neonatal and precocious deaths with seizure during life in some cases evokes a sexual chromosomal aberration in both parents or at least in the mother since deaths were more precocious, mostly neonatal in the mother's nephews and nieces. 

 Tall stature is a frequent [4] but not a constant [11] feature in 48XXYY patients. Our patient was higher than his genetic target height. Tremor has been previously reported in 47XXY, 47XYY and occurs in 71% of 48XXYY patients [4, 22]. Seizures occur in 15% of 48XXYY patients [4] and are common in patients with X chromosome anomalies.

## 4. Conclusion

Our case is a very rich observation, gathering the main typical features of 48XXYY syndrome and its associated comorbidity. Type 2 diabetes in our patient was associated with a metabolic syndrome. Our patient had retinitis pigmentosa and unilateral renal aplasia, features not reported in 48XXYY patients. We cannot rule out that these two disorders may be caused by other genetic aberrations in light of the high consanguinity rate in the patient's family. Other case reports or large studies could confirm or infirm such associations.

## Figures and Tables

**Table 1:** Biological data.

	Blood	24-h urines	Normal values
Glucose mmol/L	15		4.8–5.5
Hemoglobin g/dL	12.1		12–18
HBA1c%	11		4–5.6
Creatinin *μ*mol/L	83		53–120
Cholesterol g/L	1.6		1,5–2
HDL cholesterol g/L	0.26		0.35–0.6
Triglycerides g/L	0.9		0.40–1.50
LDL cholesterol g/L	1.16		<1.6
Calcium mmol/L, mmol/24 h	2.6	4.4	2.2–2.7, 2–7.5
Phosphore mmol/L, mmol/24 h	0.94	22	0.87–1.45, 20–33
Protides g/L, g/24 h	67	0	62–80, 0
Albumin g/L	30.5		39–46
Gamma globulin g/L	20,5		6–10
Prothrombine ratio%	78		70–100
ASAT UL/L	216		0–46
ALAT UL/L	303		0–46
GGT UL/L	53		10–46
Bilirubin *μ*mol/L	14		0–17
Creatin phosphokinase UL/L	38		25–195
Lactate deshydrogenase UL/L	236		200–400
Pyruvate *μ*mol/L	142		30–80
Lactate mmol/L	1.6		0.6–2.4
Ferritin *μ*g/L	96.2		30–350

HBA1c: glycated hemoglobin; HDL: high density lipoprotein; LDL: low density lipoprotein; ASAT: aspartate aminotransferase; ALAT: alanine aminotransferase; and GGT: gamma glutamyl transferase.

**Table 2:** Hormonal data.

	Blood	Normal values
Testosterone *μ*g/L	0.7	3–8.5
Free T4 ng/dL	0.9	0.58–1.64
TSH mUL/L	1.37	0.34–5.6
ACTH pg/mL	12	<50
Parathormone pg/mL	50	15–100

TSH: thyroid stimulating hormone; ACTH: adrenocorticotropin; and T4: thyroxine.

**Table 3:** Dynamic endocrine tests.

Test	GnRH (100 *μ*g)		ACTH (250 *μ*g)
Hormone tested	FSH (UL/L)	LH (UL/L)	Cortisol (*μ*g/L)
0′	46.4	22.09	121.6
30′	51.25	52.17	
60′	56.68	54.6	367.5
90′	58.04	44.53	

ACTH: adrenocorticotropic hormone; FSH: follicle stimulating hormone; LH: luteinizing hormone; and GnRH: gonadotropin releasing hormone.
